# Setup and Validation of Flow Cell Systems for Biofouling Simulation in Industrial Settings

**DOI:** 10.1100/2012/361496

**Published:** 2012-04-26

**Authors:** Joana S. Teodósio, Manuel Simões, Manuel A. Alves, Luís F. Melo, Filipe J. Mergulhão

**Affiliations:** ^1^LEPAE-Department of Chemical Engineering, Faculty of Engineering, University of Porto, Rua Dr. Roberto Frias s/n, 4200-465 Porto, Portugal; ^2^CEFT-Department of Chemical Engineering, Faculty of Engineering, University of Porto, Rua Dr. Roberto Frias s/n, 4200-465 Porto, Portugal

## Abstract

A biofouling simulation system consisting of a flow cell and a recirculation tank was used. The fluid circulates at a flow rate of 350 L· h^−1^ in a semicircular flow cell with hydraulic diameter of 18.3 mm, corresponding to an average velocity of 0.275 m· s^−1^. Using computational fluid dynamics for flow simulation, an average wall shear stress of 0.4 Pa was predicted. The validity of the numerical simulations was visually confirmed by inorganic deposit formation (using kaolin particles) and also by direct observation of pathlines of tracer PVC particles using streak photography. Furthermore, the validity of chemostat assumptions was verified by residence time analysis. The system was used to assess the influence of the dilution rate on biofilm formation by *Escherichia coli* JM109(DE3). Two dilution rates of 0.013 and 0.0043 h^−1^ were tested and the results show that the planktonic cell concentration is increased at the lower dilution rate and that no significant changes were detected on the amount of biofilm formed in both conditions.

## 1. Introduction

Biofilms are complex communities of surface-attached microorganisms, comprised either of a single or multiple species [1–3]. Although adhesion to a surface may look unattractive at first glance due to a more difficult access to nutrients, the fact is that in nature probably 99% or more of all bacteria exist in biofilms [4].

Despite some beneficial applications of biofilms, for instance, in industrial production of various chemicals [5–7], wastewater treatment [8], removal of volatile compounds from waste streams [9], or even energy production [10], the fact is that detrimental biofilms are far more notorious than beneficial biofilms. Negative effects of biofilm formation are often found in industrial settings where biofouling costs can represent up to 30% of the plant operating costs [11], and in the overall expenditure of industrialized countries where estimates for fouling costs (of which biofouling possibly accounts for one third) represent 0.25% of the gross national product [11].

Since beneficial and detrimental biofilms exist, it is useful to develop strategies for biofilm control that promote the formation of beneficial biofilms and delay the formation of detrimental biofilms or promote their destruction. This requires intensive studies on the mechanisms of biofilm formation and resistance and prompted the need to develop *in-vitro* platforms for biofilm studies. These platforms are artificial biofilm model systems that are easy to control and reproducible enabling a more detailed study of this phenomena.

Flow cells have been used for more than 30 years for the study of dynamic biofilms. Although different configurations have been proposed for the biofilm forming system, flow cells are often placed downstream of a biological reactor that is the source of microorganisms. In order to study biofilm formation using equilibrium conditions, it is convenient to use the effluent from a chemostat to feed the flow cell. This system provides a constant concentration of exponentially growing cells with the added advantage that the composition of the effluent stream is also constant. This system has the drawback of being limited to relatively low flow rates due to the time and expense entailed with media preparation. Furthermore, the flow rate in the flow cell is fixed by the dilution rate used on the chemostat which reduces the range of hydrodynamic conditions that can be investigated. An alternative configuration can be used where the chemostat is part of a recirculating loop that is continuously fed by a nutrient stream. This configuration can be used to achieve the high flow rates that are common in industrial processes [12]. In this case, the flow velocity in the flow cell is independent of the dilution rate of the system and higher shear rates can be achieved. In essence these systems are “chemostats with irregular geometries” [13]. In order to prevent gradient formation along the system, the recirculation flow rates must be high and the volume of the recycle loops must be minimized thus decreasing residence times. Ideally, the residence time in the entire recycle loop and flow cell should not exceed a few minutes so that the whole system can be assumed to be completely mixed [13] given the time frame of the experiments that last for several days. In industrial settings, molecules and microorganisms are transported in process streams usually under turbulent flow and, therefore, the velocity field of the fluid in contact with the microbial layer will affect biofilm structure and behaviour [14–16]. Thus, hydrodynamic conditions will determine the rate of transport of cells, oxygen, and nutrients to the surface, as well as the magnitude of the shear stress acting on a developing biofilm [17]. The importance of hydrodynamic conditions in microbial adhesion to surfaces has prompted the development of flow cell types with different designs for biofilm studies [13]. These flow cells are constructed so that a large surface area is available on which the hydrodynamic conditions remain constant for a wide range of flow velocities. One of the key design issues concerns the geometry of the flow cell and the inlet conditions which dictate the length required for flow development [18]. The hydrodynamics in flow cells can be simulated numerically using computational fluid dynamics (CFD). For turbulent flow conditions, one of the most reliable two-equation-based models to describe turbulence is the *k*-*ω* model. This model is based on two transport equations, one for the turbulent kinetic energy and another for the specific dissipation rate. The shear-stress transport (SST) version of the *k*-*ω* model [19] blends effectively the *k*-*ε* model [20] and the standard *k*-*ω* model [21] and usually leads to accurate simulations both in free stream and wall bounded flows.

The use of CFD tools is particularly useful to obtain detailed information about the flow field in complex geometries, allowing the prediction of different flow variables, such as shear and normal stresses exerted on the flow cell walls, which are highly relevant to understand their influence on the development and growth of biofilms. On the other hand, the ability to accurately simulate the entire flow field in the flow cell allows us to determine the required flow-path lengths to achieve fully developed hydrodynamic conditions, and to assess how uniform is the stress field in the flow cell walls.

In this work, we have validated results obtained using numerical simulation, by inorganic deposit formation studies and also by observation of fluid pathlines of polyvinyl chloride (PVC) particles using streak photography. The validity of chemostat assumptions was verified by residence time analysis. We have also assessed the effect of two dilution rates on *E. coli* biofilm formation under a constant turbulent flow rate.

## 2. Materials and Methods

### 2.1. Numerical Simulations

**Figure 1:**
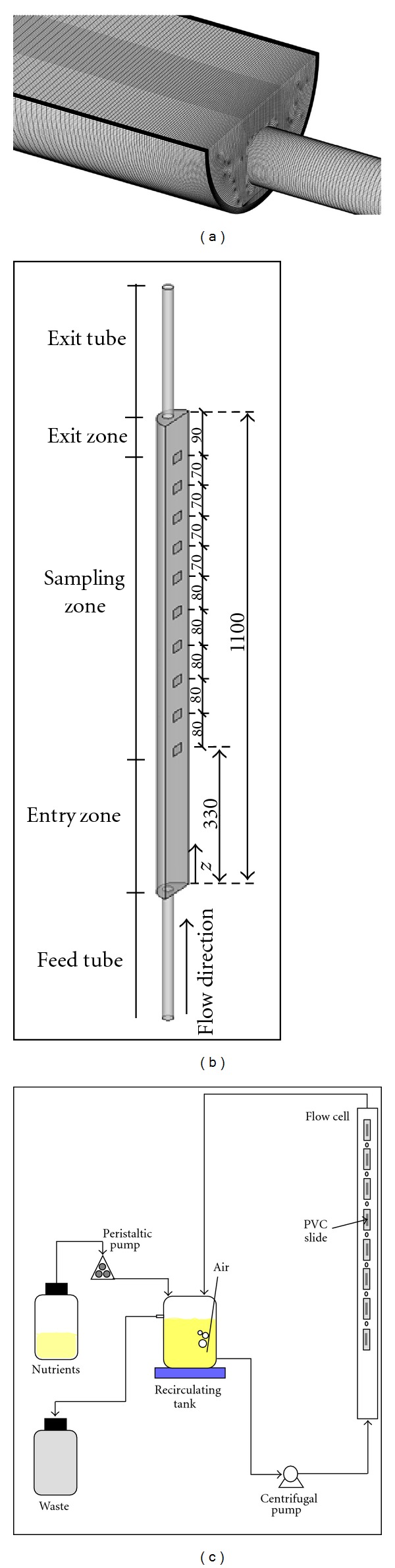
(a) Zoomed view near the entrance of the computational mesh used; (b) illustration of the flow cell and connecting tubes. Identification of the axial location of the center of the coupons (dimensions are reported in mm); (c) schematic representation of the biofilm producing flow cell system.

The Fluent CFD code (version 6.2.16, Fluent Inc.) was used in the numerical simulation of the flow field in the semicircular flow cell. The computational mesh used in the simulations was created using Gambit 2.2.30 mesh generator (Fluent Inc.) and has a total number of 1 250 472 hexahedral cells. As shown in Figure 1(a), the mesh resolution is very high, especially near the walls, hence numerical accuracy is high. The mesh generated includes the semicircular flow cell (110.0 cm length; 3.0 cm diameter), and the connecting circular tubes, both with a length of 30.0 cm and 8.0 mm internal diameter, as sketched in Figure 1(b).

To increase the numerical accuracy, the convective terms were discretized with the QUICK scheme, which is third-order accurate [22], while the pressure-velocity coupling was enforced using the PISO algorithm (Pressure-Implicit with Splitting of Operators) proposed by Issa [23]. The time integration used a fixed time step, *δt* = 0.005 s, and a second-order implicit Euler method was selected to enhance accuracy in time. Preliminary numerical tests, using different time-step values, showed that such time-step value is small enough to achieve high numerical accuracy along the oscillation cycles.

### 2.2. Flow Visualization

To complement the numerical simulations, a study of the flow patterns for a range of Reynolds numbers was performed using a long-exposure streak photography technique.

This method consists in the placement of reflective agents in the fluid. These agents were PVC spherical particles with a diameter of 50 *μ*m at a weight concentration of 100 ppm. The illumination was provided by a 635 nm laser diode equipped with a cylindrical lens (Vector, model 5200-20, 5 mW), creating a laser light sheet which illuminates a specific plane of the flow cell. The flow pattern images representing the pathlines were obtained by a digital camera (Canon EOS 30D) equipped with a macro lens (Canon EF100 mm), which was positioned perpendicularly to the light sheet.

### 2.3. Bacterial Strain and Culture Conditions

*Escherichia coli* JM109(DE3) was used in this work because this particular strain is capable of producing significant amounts of biofilm [24]. A starter culture was prepared by inoculation of 300 *μ*L of a glycerol stock (kept at −80°C) to a total volume of 0.5 L of inoculation media, as previously described by Teodósio et al. [24]. Optical density (O.D.) measurement at 610 nm was used to monitor cell growth and when the O.D. reached 1, cultures were used to inoculate the recirculating tank.

In order to obtain two different dilution rates, the same feed flow rate of 0.0252 L*·*h^−1^ was used in two systems where the only difference was the volume of the recirculating tank (all the remaining system components were maintained). Thus, a recirculating tank of 5 L (corresponding to a total system volume of 5.92 L–Table 1(a)) was used to obtain a dilution rate of 0.0043 h^−1^, and a recirculating tank of 1 L (corresponding to a total volume of 1.92 L–Table 1(b)) was used to obtain a dilution rate of 0.013 h^−1^. For inoculation, the 1 L recirculating tank contained 0.5 L of sterile water and the 5 L recirculating tank contained 2.5 L of sterile water. Recirculating tank feeding started 5 h after inoculation at a flow rate of 0.0252 L*·*h^−1^ with the nutrient media consisting of 0.55 g·L^−1^ glucose, 0.25 g·L^−1^ peptone, 0.125 g·L^−1^ yeast extract, and phosphate buffer (0.188 g·L^−1^ KH_2_PO_4_ and 0.26 g*·*L^−1^ Na_2_HPO_4_), pH 7.0.

### 2.4. Biofilm Producing System

A reactor system consisting of a recirculating tank, one vertical flow cell built in Perspex, peristaltic and centrifuge pumps, was used as described by Teodósio et al. [24] (Figure 1(c)). PVC slides (2 cm × 1 cm) are glued onto the removable coupons of the flow cell and are in contact with the bacterial suspension circulating in the system. PVC was chosen because it is commonly found in piping systems, although the slide material can be changed to simulate other surfaces. The recirculating tank, for planktonic cell growth, was built with a cooling jacket to enable temperature control. Temperature was kept constant at 30°C using a recirculating water bath in order to simulate conditions commonly found on industrial settings.* E. coli* cells were grown by recirculating the bacterial suspension during 13 days at a flow rate of 350 L*·*h^−1^. Turbulent flow with Reynolds number of 6 290 was used for biofilm formation which is also typical for many industrial processes.

### 2.5. Sampling and Analysis

For biofilm sampling, the system was stopped to allow coupon removal and carefully started again maintaining the same flow conditions, as described by Teodósio et al. [24]. Biofilm wet weight was determined by weighing the coupon immediately after retrieval from the flow cell and subtracting the weight of the empty coupon that had been determined during system assembly. Biofilm thickness was determined using a digital micrometer (VS-30H, Mitsubishi Kasei Corporation) [24].

The optical density and glucose concentration were determined in the recirculating tank. Optical density was measured using a Spectrophotometer at 610 nm (T80 UV/VIS Spectrometer/PG Instrument, Ltd.). Glucose quantification was performed by dinitrosalicylic colorimetric method (DNS) adapted to a microtiter plate format as described by Teodósio et al. [24]. Glucose consumption values were obtained from a mass balance, by multiplying the glucose concentration difference (feed minus concentration on the tank) by the feed flow rate (0.0252 L·h^−1^).

### 2.6. Deposit Formation by Inorganic Material

Kaolin powdered particles, with a distribution of diameters ranging between 5 and 10 *μ*m [25], were used in an experiment to verify the formation of inorganic deposits on the biofilm producing system. The experimental setup was properly cleaned (with bleach) and sterile water was introduced in the system. A kaolin suspension with the final concentration of 2.0 g*·*L^−1^ was prepared and introduced in the system after the total removal of the sterile water. The system operated continuously at a flow rate of 350 L·h^−1^ for one week.

### 2.7. Statistical Analysis

Biofilm formation results originated from three independent experiments for each dilution rate condition. Paired *t*-test analyses were performed to estimate whether or not there was a significant difference between the results. Each time point was evaluated individually using the three independent results obtained in one condition and the three individual results obtained on the other condition. When a confidence level greater than 95% (*P* < 0.05) was obtained, these time points were marked with an *.

## 3. Results

### 3.1. Numerical Simulation of the Flow

In the numerical simulations, a recirculation flow rate of 350 L·h^−1^ was considered, at 30°C, corresponding to a Reynolds number of 6 290. The Reynolds number is here defined as

(1) Re=ρUDhμ,

where *ρ* and *μ* are the density and dynamic viscosity of the fluid, respectively, *U* is the average velocity in the flow cell, and *D*_*h*_ is the hydraulic diameter of the semicircular flow cell (*D*_*h*_ = *πD*/(2 + *π*) = 1.83 cm) of diameter *D*. In the inlet and outflow circular tubes connecting the flow cell (of diameter 8 mm), the Reynolds number was higher (*Re* = 19 300), therefore, the flow was turbulent in the full flow domain.

**Figure 2:**
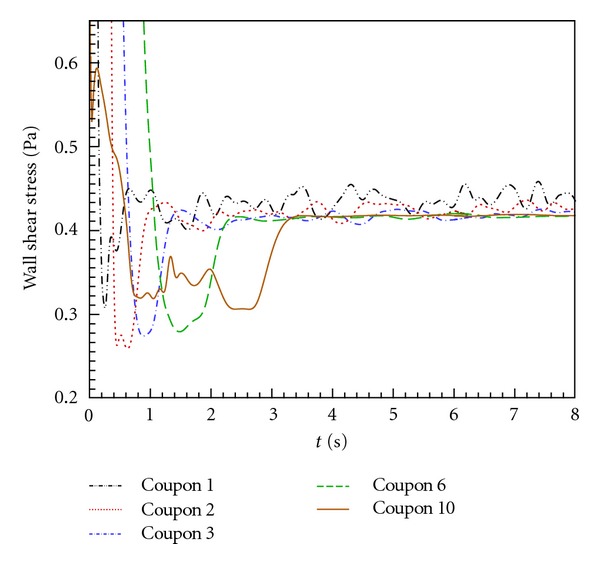
Time evolution of the magnitude of the wall shear stress acting on coupons 1, 2, 3, 6, and 10.

Figure 2 illustrates the time evolution of the average wall shear stresses acting on coupons 1, 2, 3, 6, and 10. Coupons 1–3 were selected to represent locations where flow is still spatially developing, while coupon 10 (last) was selected to show that, despite being close to the exit of the flow cell, the local flow was not influenced by the abrupt contraction to the downstream exit tube. Coupon 6 was representative of fully developed flow conditions and shows clearly that coupons 3–10 were also representative of fully developed flow conditions, to within a variation below 5%. In coupons 1 and 2, there were still some entrance effects, and the transients were more intense, nonetheless the variation of the average wall shear stress was still below 5%, an acceptable variation to still consider those first two coupons as representative of the fully developed flow conditions and accept them as reliable for monitoring biofilm growth in the semicircular flow cell.

Figure 2 also illustrates that after a transient period of about 1–3 s, depending on the coupon location, the flow patterns oscillated periodically with a dominant frequency of about 3 Hz. The average values of the wall shear stress on each coupon were 0.440, 0.425, 0.417, 0.417, and 0.417 Pa for coupons 1, 2, 3, 6, and 10, respectively. The amplitude of wall shear stress oscillations was higher in coupons 1 and 2 (±0.02 and 0.015 Pa, resp.), but overall the average shear stresses were very similar on all coupons. It is important to bear in mind that these small oscillations along time are not important, and the average values should be considered in the analysis given the time scale of the biofilm experiments, of the order of several days.

**Figure 3:**
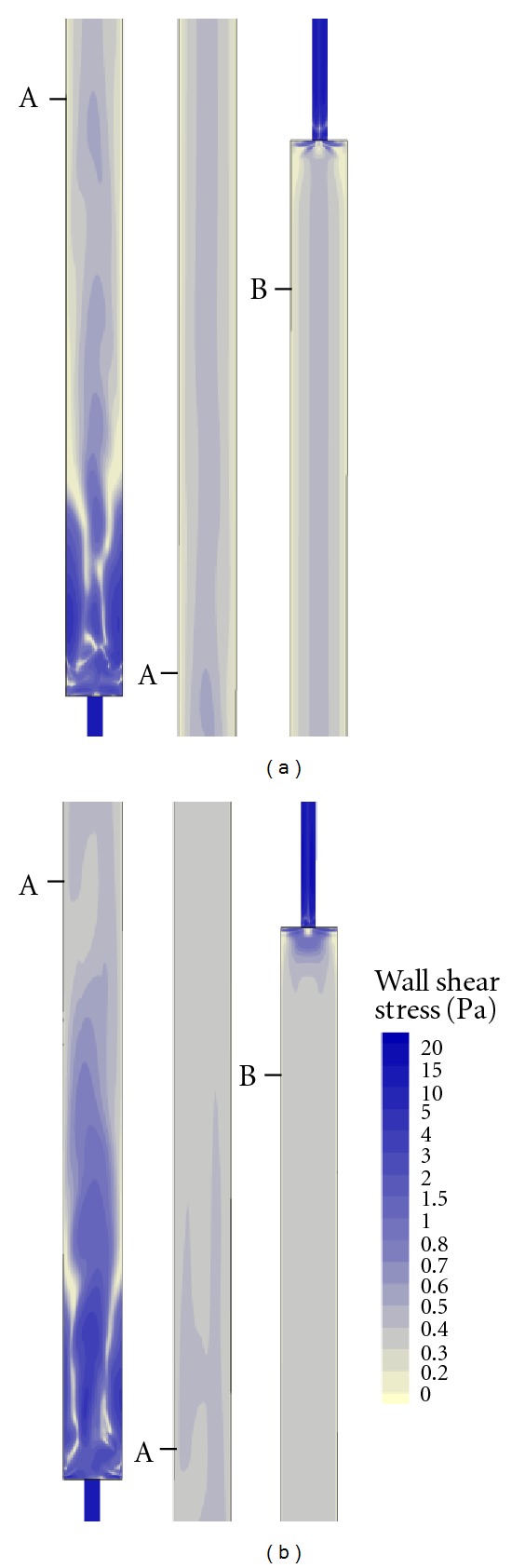
Contour plots of the instantaneous magnitude of the wall shear stress field on the (a) planar and (b) circular walls. In both cases the entry zone, fully developed flow region and exit zones are shown. The center of the first and last coupons is identified by letters A and B, respectively. The flow direction is from bottom to top.

Figure 3 shows instantaneous contour plots of the wall shear stress acting on both the planar and round walls of the flow cell, and on the entry and outflow circular tubes. The wall shear stresses were similar in all coupons, except for the first ones, where an increase below 5% was observed. Also, it is relevant to emphasize that despite the transverse variation of the shear stress observed in the flat and rounded walls, with minimum values on the intersection of both walls, the shear stresses predicted in the central part of the flat wall, where the coupons are located, were very similar to the predicted shear stresses on the curved wall, thus demonstrating that the average wall shear stresses acting on the coupons on the planar walls are representative of the shear stresses acting on the curved wall. This is an indication that the semicircular cell is a good representation of the wall shear stresses observed in circular tubes, which are typically used in industrial piping systems.

**Figure 4:**
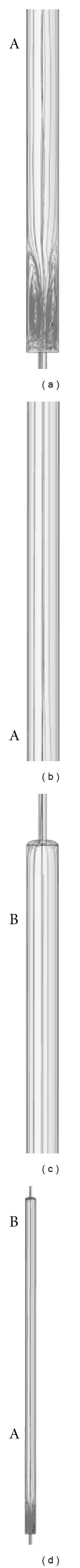
Instantaneous streamlines on the (a) entrance, (b) middle, and (c) exit regions of the semicircular flow cell. In (d) a global view is presented. The flow direction is from bottom to top, and the views illustrated are through the planar wall. Point A represents the position of the center of coupon 1 and point B the position of the center of coupon 10.

Figure 4 displays a frontal view through the planar wall of the instantaneous streamlines predicted numerically for the same instant illustrated in Figure 3. The results illustrate the long inlet developing length, and the negligible influence of the outflow boundary, justifying the use of a longer entry region to guarantee that in the first coupon the flow is nearly fully developed.

**Figure 5:**
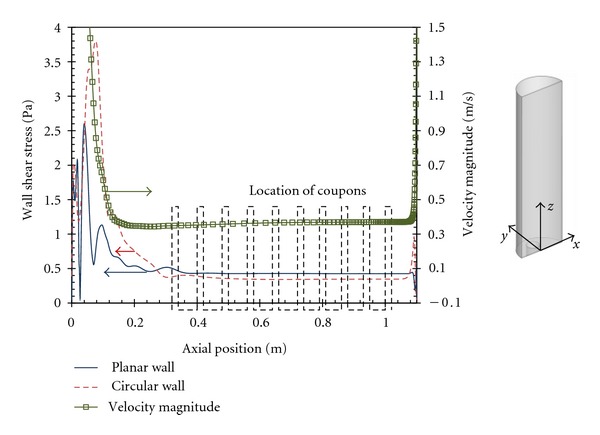
Streamwise variation of the magnitude of the wall shear stress along the center of the planar (*x* = 0, *y* = 0) and round walls (*x* = 0, *y* = 0.015 m). The velocity magnitude profile is also included, but along the center of the semicircular flow cell (*x* = 0, *y* = 0.0075 m).

Figure 5 presents streamwise plots of the instantaneous magnitude of the wall shear stress along the center of the planar and circular walls. To better assess the entry length necessary to achieve fully developed flow conditions, the streamwise variation of the velocity magnitude along the center of the semicircular duct is also included. The results confirm unequivocally that even the first coupon was already under quasi-fully developed flow conditions. The difference in the wall shear stresses between the center of the planar and circular walls is small, showing that the wall shear stress on the planar wall is representative of the wall shear stress on the circular wall.

### 3.2. System Validation

**Figure 6:**
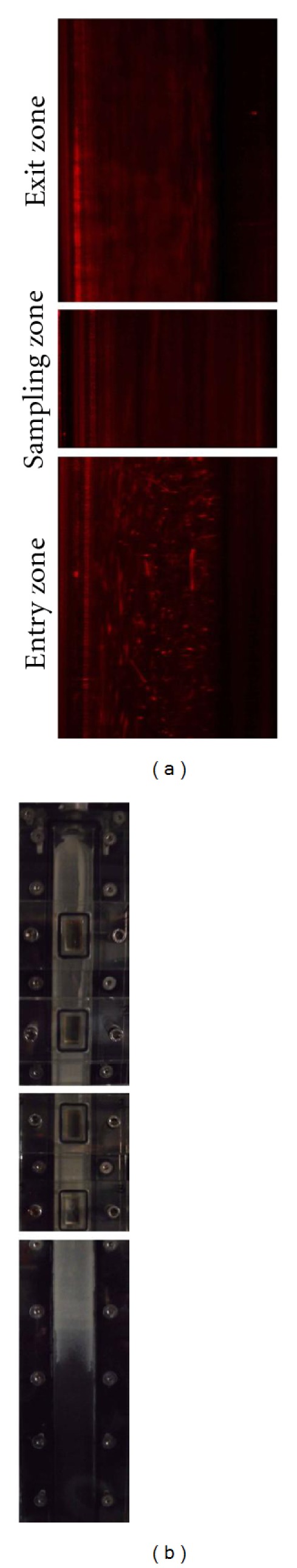
Flow visualization using tracer particles illuminated with a laser diode (a) and inorganic deposit formation (b) at three different zones of the flow cell: entry, sampling, and exit.

After numerical simulation of the hydrodynamic conditions by CFD, the flow was visualized using tracer particles illuminated with a laser diode. Photographs taken during this experiment are shown on Figure 6(a) where it is possible to verify that particles follow a quasilinear trajectory on the sampling area confirming numerical simulation results. This flow behavior is very different from the entry zone where significant vortices can be observed. Perturbations on the exit zone are not very clear from the tracer particles experiment and also confirm the results obtained by numerical simulation.

In order to further assess the hydrodynamic effects during biofilm formation, a suspension of kaolin particles (diameter 5–10 *μ*m; density 2.6 g·cm^−3^) was used and inorganic deposits were formed on the flow cell system (using the same flow rate conditions used during biofilm formation). Although the physical properties of kaolin particles are different from bacterial cells, these particles are highly adherent to Perspex surfaces as it can be seen on Figure 6(b). This figure shows that there are regions in the entry and exit zones where adhesion is prevented possibly due to the higher shear stresses generated in those regions and the more intense velocity fluctuations in those locations. The lengths of these deposit free zones are consistent with the CFD predictions presented on Figures 3, 4, and 5 which is an additional indication that the output of the numerical simulation is valid for the real life conditions used on this experiment. Indeed, by observing deposit formation (Figure 6(b)), it was possible to measure the flow cell entry and exit zone lengths: 18 cm and 0.5 cm, respectively. Deposit formation was observed on the entire flow cell, except for the entry zone (up to 18 cm from the inlet) and very close to the outlet (0.5 cm). These observations agree with the results presented in Figure 4, where a long entry zone region is required to guarantee fully developed flow in the first coupon area. The negligible influence of the outflow boundary was also confirmed. One of the most significant advantages in using a recirculating system (where the flow cell is fed by the effluent of a chemostat and the flow is recirculated after passage back to the chemostat) is the possibility of adjusting the flow rate on the flow cell independently of the nutrient flow rate in order to achieve higher fluid velocities [13]. In order to guarantee that estimates of activity or the microbiology of the attached cells and the planktonic cell population in the effluent truthfully reflect the processes occurring on the system, chemostat assumptions are used and, therefore, the system is assumed to be completely mixed [13]. If the flow rate in the recirculation tube is low, significant gradients may build up along the length of the recycling tube, in the recirculating tank or even in the flow cell and, therefore chemostat kinetics cannot be used. In order to have a well-mixed system, the residence time in each of the components of the system must be minimized (e.g., by using the minimum length of connecting tube) and the total residence time in the system should not exceed a few minutes [13].

In order to obtain two different dilution rates, two different reactors of 1 L and 5 L were used in independent experiments and the feed flow rate and the recirculation rates were maintained (feed flow rate 0.0252 L·h^−1^ and recirculating rate 350 L·h^−1^). The residence time analysis in both systems shows that the total residence time is equal to or less than one minute (Tables 1(a) and 1(b)). This indicates that the mass transfer processes controlled by the hydrodynamics of the system are much faster than the metabolic response by the cells and that the system can be considered a well-mixed chemostat with an irregular geometry [13].

### 3.3. Biofilm Formation

Biofilm formation by *E. coli* JM109(DE3) was assessed on a flow cell operated at a Reynolds number of 6 290 during 13 days. An experimental apparatus [24] including a recirculating tank system allowed biofilm growth under well-defined and controlled conditions and biofilm sampling for analysis. Two dilution rates (0.013 and 0.0043 h^−1^) were tested using a glucose concentration of 0.55 g·L^−1^ on the feed stream.

**Figure 7:**
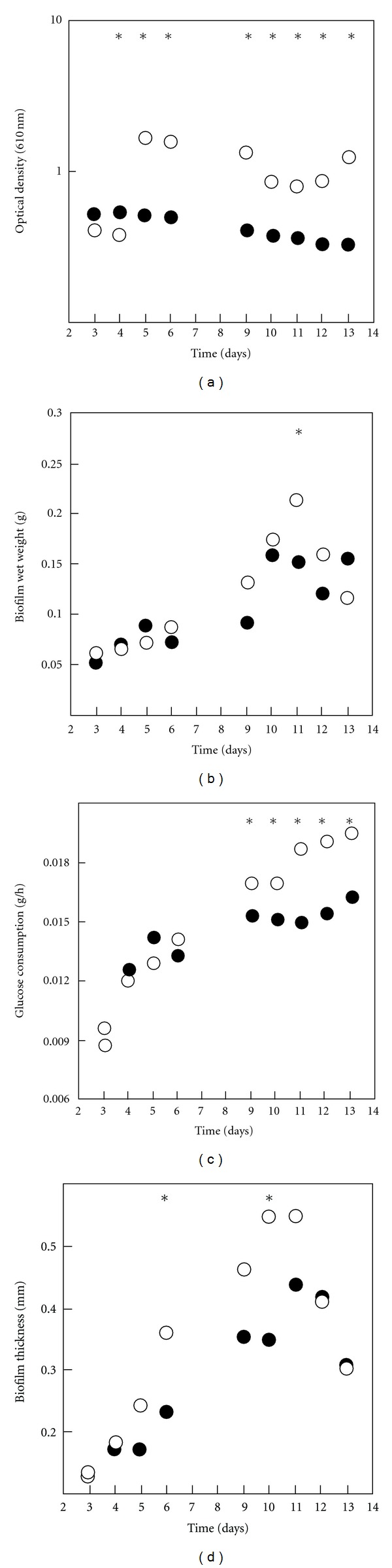
Time-course evolution of planktonic and biofilm assayed parameters for *E. coli* JM109(DE3). Closed circles: dilution rate of 0.013 h^−1^, open circles: dilution rate of 0.0043 h^−1^. (a) Planktonic optical density (610 nm), (b) glucose consumption in the system (g·h^−1^), (c) biofilm wet weight (g) and (d) Biofilm thickness (mm). Results are an average of those obtained in three independent experiments for each condition. Time points marked with an * are those for which a statistically significant difference was obtained (for a confidence level greater than 95%).

Figure 7 shows the results obtained for the planktonic and biofilm parameters analyzed during the experiment. In order to evaluate planktonic cell growth, optical density (O.D.) measurements were performed in the recirculating tank (Figure 7(a)). Glucose consumption profiles (Figure 7(b)) were determined by sampling the recirculating tank and establishing a mass balance. Biofilm samples were retrieved from the coupons for quantification of wet weight (Figure 7(c)) and thickness (Figure 7(d)). The analysis of planktonic optical density (Figure 7(a)) shows a distinct behavior (*P* < 0.05) between cells grown under different dilution rates. With the exception of days 3 and 4, higher concentrations of planktonic cells were obtained for the lower dilution rate. For the higher dilution rate, planktonic cell concentration remained constant throughout the experiment whereas for the lower dilution rate cell, concentration increased between days 4 and 5, then equilibrium was reached.

Glucose consumption increased during the experimental time for both dilution rates (Figure 7(b)). The profiles are very similar (*P* > 0.05) until day 9 and then glucose consumption in the lower dilution rate was higher (*P* < 0.05) which is consistent with an increased planktonic cell concentration in these conditions. Concerning biofilm formation results, similar profiles were observed for wet weight and thickness (Figure 7(c) and 7(d)) with thicker biofilms obtained in the lower dilution rate between days 6 and 11. Previous studies by Wijeyekoon et al. [26], using microscopic methods, showed that an increase in nutrient loading led to the formation of compact and thinner biofilms which is consistent with our results. The wet weight of the biofilms obtained in both conditions increases until days 10-11 and then decreases probably due a predominance of sloughing/detachment phenomena when compared to the attachment of new biofilm cells. The biofilm thickness results (Figure 7(d)) are consistent with the wet weight analysis and similar values are obtained for both parameters at the end of the experiment (*P* > 0.05).

Although it has been shown that high glucose concentrations can inhibit biofilm formation [27, 28], contradictory results were also reported [29] indicating that higher glucose concentrations may also be beneficial. Additionally, we have previously used higher dilution rates (of 0.165 h^−1^) for biofilm formation with this same strain (not shown) and, therefore, we know that we were not exceeding the critical dilution rate in this situation. In the present conditions, an increase in dilution rate is not favorable for planktonic cell development and has no significant impact on the amount of biofilm formed. Apparently, the higher dilution rate may exceed the maximum growth rate of planktonic cells in these conditions. Arguably, washout is not observed due to the dynamic process of biofilm detachment and sloughing events which may be a source for planktonic cells. However, since the nutrient loading is increased at higher dilution rates but the hydrodynamic conditions were maintained (*Re* = 6 290), we hypothesize that in these conditions the hydrodynamics are more important than nutrient availability in controlling the amount of formed biofilm as observed by independent groups [30, 31] working with mixed species biofilms.

## 4. Conclusions

A flow cell system was used to study the effects of the dilution rate on biofilm formation using *E. coli* JM109(DE3). The hydrodynamic conditions on the flow cell were simulated using CFD and it was shown that under the flow rate conditions that were used, a fully developed flow was obtained on the sampling section. It was also demonstrated that the entry zone had a long enough distance to allow flow development and that the effect of the sudden contraction on the exit zone was negligible. The validity of the models used for the simulation was confirmed by flow visualization and particle deposition experiments. Besides the hydrodynamic validation, the validity of chemostat assumptions was also verified by residence time analysis. Altogether these results show that this biofilm forming system comprising the flow cell and the recirculation tank is valid for biofilm formation studies at these flow rates. Biofilm formation assays showed that the lower dilution rate favored planktonic growth and biofilm thickness although the mass of biofilm formed was similar in both conditions. Although these biofilm experiments were performed using a particular *E. coli* strain, we have shown by time-residence analysis, particle deposition, and flow visualization experiments that this experimental setup and flow simulation by CFD are robust enough to be used with other biofilm producing bacteria.

## Figures and Tables

**Table 1a:** (a)

Component	Volume (mL)	Residence time (s)
Recirculating tank	5000	51.4
Flow cell	300	3.1
Recirculating tubing	620	6.4
Whole system	5920	60.9

**Table 1b:** (b)

Component	Volume (mL)	Residence time (s)
Recirculating tank	1000	10.3
Flow cell	300	3.1
Recirculating tubing	620	6.4
Whole system	1920	19.7

## Nomenclature

*D*: Diameter, m
*D*_*h*_: Hydraulic diameter, *πD*/(2 + *π*), m
*μ*: Fluid dynamic viscosity, Pa*·*s
*Re*: Reynolds number, *Re* = *ρUD*_*h*_/*μ*, dimensionless
*ρ*: Fluid density, kg·m^−3^
*U*: Average velocity, m·s^−1^.
